# Standardizing Visual Control Devices for Tsetse Flies: West African Species *Glossina tachinoides*, *G. palpalis gambiensis* and *G. morsitans submorsitans*

**DOI:** 10.1371/journal.pntd.0001491

**Published:** 2012-02-14

**Authors:** Jean-Baptiste Rayaisse, Thomas Kröber, Andrew McMullin, Philippe Solano, Steve Mihok, Patrick M. Guerin

**Affiliations:** 1 Centre International de Recherche-Développement sur l'Elevage en zone Subhumide (CIRDES), Bobo-Dioulasso, Burkina Faso; 2 Institute of Biology, University of Neuchâtel, Neuchâtel, Switzerland; 3 Institut de Recherche pour le Développement (IRD), Unité Mixte de Recherche (UMR) 177 - Centre de Coopération Internationale en Recherche Agronomique pour le Développement (CIRAD), CIRDES, Bobo-Dioulasso, Burkina Faso; 4 Russell, Ontario, Canada; International Centre of Insect Physiology and Ecology, Kenya

## Abstract

Here we describe field trials designed to standardize tools for the control of *Glossina tachinoides*, *G. palpalis gambiensis* and *G.morsitans submorsitans* in West Africa based on existing trap/target/bait technology. Blue and black biconical and monoconical traps and 1 m^2^ targets were made in either phthalogen blue cotton, phthalogen blue cotton/polyester or turquoise blue polyester/viscose (all with a peak reflectance between 450–480 nm) and a black polyester. Because targets were covered in adhesive film, they proved to be significantly better trapping devices than either of the two trap types for all three species (up to 14 times more for *G. tachinoides*, 10 times more for *G. palpalis gambiensis*, and 6.5 times for *G. morsitans submorsitans*). The relative performance of the devices in the three blue cloths tested was the same when unbaited or baited with a mixture of phenols, 1-octen-3-ol and acetone. Since insecticide-impregnated devices act via contact with flies, we enumerated which device (traps or targets) served as the best object for flies to land on by also covering the cloth parts of traps with adhesive film. Despite the fact that the biconical trap proved to be the best landing device for the three species, the difference over the target (20–30%) was not significant. This experiment also allowed an estimation of trap efficiency, i.e. the proportion of flies landing on a trap that are caught in its cage. A low overall efficiency of the biconical or monoconical traps of between 11–24% was recorded for all three species. These results show that targets can be used as practical devices for population suppression of the three species studied. Biconical traps can be used for population monitoring, but a correction factor of 5–10 fold needs to be applied to captures to compensate for the poor trapping efficiency of this device for the three species.

## Author Summary

Tsetse flies transmit trypanosomes that cause sleeping sickness in humans and ngana in animals in sub-Saharan Africa. These diseases remain an intractable burden on human health and livestock production on the continent. Visual devices made of fabrics and impregnated with insecticide have the potential for controlling tsetse and preventing disease transmission as regular removal of a small percentage of the vector population can provide effective control. A variety of devices, notably biconical and monoconical traps and targets, have been used in previous control programmes against tsetse in West Africa. In this study we set out to determine which is the optimal device for control of three tsetse fly species, *Glossina tachinoides*, *G. palpalis gambiensis* and *G. morsitans submorsitans*, in the region. Using the same colourfast fabrics in all devices we show that there is no significant difference between biconical traps and targets in their ability to induce landings by the three species. We conclude that the simpler target would appear to be the most cost-effective insecticide-impregnated device to use in control programmes against these tsetse species. We also provide an estimate of the efficiency of the biconical and monoconical traps for the three species.

## Introduction

Tsetse flies (Diptera: Glossinidae) transmit trypanosomes that cause sleeping sickness in humans (Human African Trypanosomiasis) and nagana in animals (Animal African Trypanosomiasis) in sub-Saharan Africa. These diseases are an intractable burden on human health and livestock production on the continent. Many tsetse fly species are attracted to large blue and black objects; hence trapping devices made with phthalogen blue cloth with peak reflectance at 465 nm and good colour fastness have been the most effective [1]–[3]. Trapping devices have the potential for controlling tsetse flies which are peculiar among insect vectors in that they are larviparous. This means that investment by females in only a few progeny results in a low rate of population growth. Hence, sustained removal of just a small percentage of a population can provide effective control [4]. Traps have been used for sampling flies since the early 1900s, with the first readily deployable devices for control developed in the 1980s, e.g. for West African species, the biconical [5] and monoconical traps [6], and simple cloth targets [2]. Responses to these devices nevertheless vary between species and in the presence of odour baits [7]. Tsetse flies will only land on or be caught in devices of the right colour (for example visual sensitivity in *G. morsitans morsitans* peaks at 365 nm with a second plateau in the blue part of the spectrum [8]), contrast, texture, size and shape.

In this study we describe field trials to determine optimal traps and targets for three key tsetse in West Africa, namely *Glossina tachinoides* (Westwood) and *G. palpalis gambiensis* (Vanderplank) belonging to the palpalis species group and *G. morsitans submorsitans* (Newstead) in the morsitans group. Trials were based on existing knowledge of practical trap/target/bait technology with a view to standardizing tools for area-wide control of these vectors. We also compare the efficacy of selected well-characterized fabrics in all devices and present simple methods to compare the efficiency of traps and targets at the remote field sites where these vectors occur.

## Materials and Methods

### Study sites

Studies on *G. tachinoides* and *G. morsitans submorsitans* were conducted over two years along the Comoe river at Folonzo (09° 549′ N, 04° 369′ W), Comoe province, southern Burkina Faso. The area receives an annual rainfall of 1100 mm. Studies took place early in the dry season in December 2009 and November 2010 when fly numbers are highest. The site is in a protected Sudanese gallery forest with some wildlife, e.g. warthogs (*Phacochaerus aethiopicus*), hippopotamus (*Hippopotamus amphibus*), monitor lizards (*Varanus niloticus*) and hartebeest (*Alcelaphus buselaphus*). Studies on *G. palpalis gambiensis* were conducted at the end of the wet season in October at two other sites. In 2009 experiments were conducted at Solenzo (12° 149′ N, 04° 239′ W), Banwa province, western Burkina Faso along the Mouhoun river, and in 2010 in Kartasso (11°18′ N, 5°27′ W ), Kénédougou province in western Burkina Faso along the river Pindia (a tributary of the Mouhoun). The habitat along the river is Sudano-Guinean gallery forest [9], which is favourable for this species. The forest is heavily degraded elsewhere due to expansion of agriculture. Hosts in the area include humans, cattle, goats and pigs. Climatic conditions are similar to those along the Comoe River, with an annual rainfall of 1000 mm.

### Catching devices, materials and baits

Three catching devices were tested: standard biconical [5] and monoconical (Vavoua type) traps [6], and a square cloth target (1 m^2^, equal vertical rectangles of blue and black). Three different blue fabrics were tested: C180 Azur 623 phthalogen blue 100% cotton, 180 g/m^2^, TDV, Laval, France (reflectance spectral peak at 460 nm as measured with a Datacolor Check Spectrophotometer, Datacolor AG, Dietlikon, Switzerland) and referred to here as the standard fabric; S250 Azur 023 phthalogen blue 65% cotton/35% polyester, 250 g/m^2^, TDV France (peak at 450 nm); turquoise blue Q10067 65% polyester/35% viscose, 234 g/m^2^ Sunflag, Nairobi, Kenya (peak at 480 nm). One black fabric (Q15093 100% polyester, 225 g/m^2^, Sunflag, Nairobi) was used for all devices.

A 1∶4∶8 mixture of 3-n-propylphenol (P), 1-octen-3-ol (O), and p-cresol (C) (Ubichem Research LTD, Budapest, Hungary with a global purity of up to 98%) was used as the attractant [10] for comparing baited devices. Sachets made of 500 gauge/0.125 mm polyethylene containing 3 g of the mixture were placed below the catching devices, 10 cm above the ground, alongside a 250 ml bottle buried up to the shoulders containing acetone (A) with a 2 mm aperture in the stopper. This combination is termed the POCA bait.

To monitor the numbers of tsetse landing on targets, 1 m^2^ one-sided sticky adhesive film (30 cm wide rolls; Rentokil FE45, UK) was rolled around both sides of the 1 m^2^ targets. This film was attached with paper clips and clothes pegs to the cloth component of traps in some experiments to enumerate flies that land on traps but may not be captured. To assess any influence of adhesive film on landing responses, the number of flies attracted to unmodified targets was compared to targets covered with adhesive film by using an electric grid (E) of fine electrocuting copper wires (spaced 8 mm apart) mounted in front and behind the non-sticky targets, [11]. A potential difference of 40 KV was applied between adjacent wires and tsetse flies that landed on the E-target were electrocuted and fell into a tray (3 cm deep) of soapy water. The electrocuting wires are considered to be invisible to tsetse [11], [12].

### Experimental design

#### Best trapping device and blue material

To assess which was the best catching device and the most attractive blue material, a six-day experiment was carried out to compare six devices in a 6×6 Latin square design of days×sites×treatments, with 3 simultaneous replicates. Randomization was set up using design.lsd in the package *agricolae* [13], R version 2.13.0 [14]. Trap sites were always >100 m apart and flies from each device were counted after 24 hours at each position. The six devices and blue materials tested were: biconical traps in standard blue cotton, phthalogen blue cotton/polyester or turquoise blue polyester/viscose; monoconical traps in standard blue cotton or phthalogen blue cotton/polyester, and a target in standard blue cotton covered with one-sided sticky film. The 6-device experiment was repeated using the POCA bait after the unbaited trial was completed in the same general area, with trapping positions >200 m apart. The objective was to determine whether baiting changed the performance ranking of the devices/fabrics.

#### Comparing traps versus targets as landing devices

To assess the efficiency of 3-d traps versus 2-d targets as landing devices, catches in biconical and monoconical traps with sticky adhesive film on the cloth component were compared to targets covered with adhesive film. All catching devices were made of standard phthalogen blue cotton. Flies caught in the cage of the traps were not included in the total for this comparison. It was only practical to place adhesive film on the outer blue cloth of the biconical trap. This gave a total sticky surface area capable of trapping flies of 0.7 m^2^ (the blue surface without the 4 holes) on the biconical trap, of 0.9 m^2^ on the blue and black hanging portions of the monoconical trap and of 2 m^2^ for the two faces of the target. Biconical and monoconical traps not treated with the adhesive film were included as controls to estimate trap efficiency. For *G. tachinoides* and *G. morsitans submorsitans*, a 10-day experiment was carried out to compare the five devices in a 5×5 Latin square of days×sites×treatments in four replicates (replicates 3 and 4 were repeated in the same trapping positions as replicates 1 and 2). For *G. palpalis gambiensis*, a 15-day experiment was conducted using the same devices in a 5×5 Latin square of days×sites×treatments in three replicates, repeated one after another in the same trapping positions over 15 days. The trapping positions were always >100 m apart and flies of each sex from each device were counted after 24 hours at each position.

#### Testing adhesive film

To assess whether the addition of the adhesive film could affect the attraction of tsetse to a catching device, a comparison was made between catches of tsetse attracted to a cloth target with no film applied and targets covered on both sides by the adhesive film with the sticky side inwards. The two types of target were placed within electric grids (above), facing E-W, perpendicular to the river, and the experiments were conducted following a 2×2 Latin square design of days×sites×treatments, with two replicates, over 10 days. The experiments were carried out simultaneously from 8:00 am to 12:00 noon each day, to give 80 hours of observation per treatment. Trapping positions were always >100 m from one another.

### Statistical analysis of data

Data were analyzed using a general linear model (GLM) in R version 2.13.0 [14], including the following additional packages: MASS [15] and multcomp [16]. Analysis was performed on log (x+1) transformed data including day and position as additional explanatory parameters and Tukey contrasts were calculated to compare treatments. Unless otherwise specified, results are presented as detransformed means. *G morsitans submorsitans* is not mentioned where captures were too low for meaningful analysis.

## Results

### Best trapping device and blue material

When unbaited, the target covered with adhesive film was the best device for trapping *G. tachinoides*, *G. palpalis gambiensis* and *G. morsitans submorsitans*. Targets captured 4–5 times more *G. tachinoides* than the biconical traps and 9–10 times more flies than the monoconical traps (P≤0.001; Table 1). Target captures for *G. palpalis gambiensis* were 6–7 times higher than for biconical traps and 12–14 times higher than for monoconical traps (P≤0.001; Table 1). For *G. morsitans submorsitans*, targets captured 5.5–6.5 times more flies than the biconical and monoconical traps (P≤0.001; Table 1). The trapping rate (as measured from mean daily catches) of the biconical trap was twice that of the monoconical trap made of the same material for *G. tachinoides* and *G. palpalis gambiensis*; differences were significant in all but one case for each species (P<0.05; Table 1). In contrast, for *G. morsitans submorsitans* there was little difference between the performance of the biconical and monoconical traps. There was no difference between the performance of traps made from different blue cloths for any species (P>0.05; Table 1). Sex ratios were similar on the different devices for the three species.

**Table 1:** Detransformed mean daily catches of *G. tachinoides*, *G. palpalis gambiensis*, and *G. morsitans submorsitans* with unbaited and POCA-baited trapping devices made of different blue fabrics.

		*G. tachinoides*		*G. p. gambiensis*		*G. morsitans submorsitans*	
		Mean daily catch		Mean daily catch		Mean daily catch	
Device	blue material	unbaited	POCA baited	unbaited	POCA baited	unbaited	POCA baited
Biconical	standard	27.4^a b^	32.1^a^	5.1^a^	4.0^a^	2.3^a^	8.5^a^
Biconical	S250	32.1^a^	18.9^a^	5.1^a^	2.5^a b^	2.1^a^	8.6^a^
Biconical	turquoise	31.8^a^	32.5^a^	4.8^a b^	3.2^a^	2.1^a^	9.6^a^
Monoconical	standard	14.9^c^	7.1^b^	2.4^c^	2.0^b c^	2.0^a^	4.1^b^
Monoconical	S250	16.4^c b^	5.7^b^	2.7^b c^	1.5^b c^	1.9^a^	3.7^b^
Target	standard	146.9^d^	323.8^c^	33.8^d^	9.3^d^	12.8^b^	80.6^c^

Means followed by the same letter within columns are not significantly different (ANOVA followed by Tukey post hoc test, P = 0.05). See text for details on blue fabrics and POCA bait.

### Best landing device

**Figure 1:**
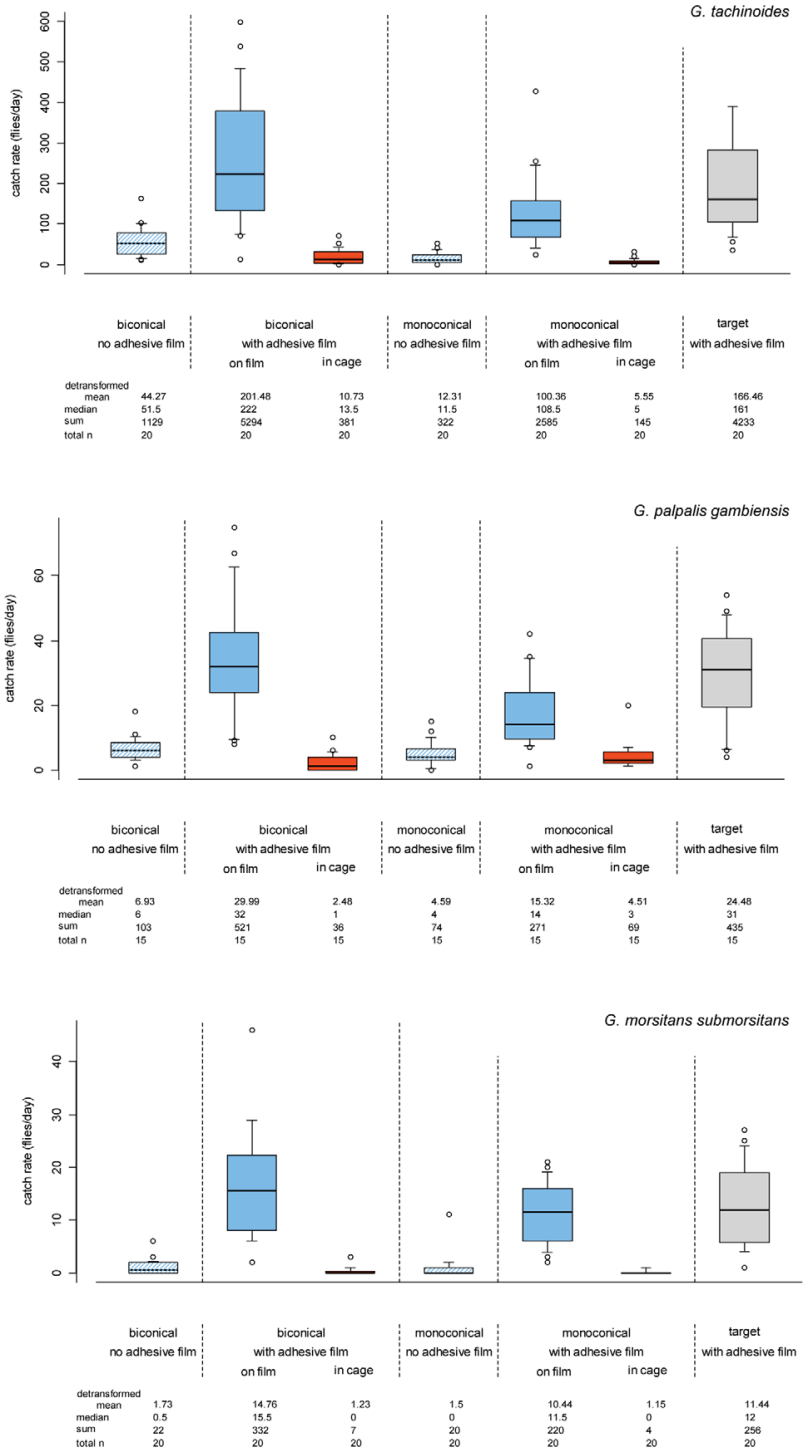
Daily catch rates of *G. tachinoides* (top), *G. palpalis gambiensis* (middle) and *G. morsitans submorsitans* (bottom) by traps and a target. The target and the cloth portions of traps were covered with adhesive film to compare the propensity of flies to land on these different devices. Catch rates of such traps were separated into fly catches on the cloth part and those trapped in the cage of the trap. Biconical and monoconical traps not treated with the adhesive film were included as controls. The limits of the boxes indicate the twenty-fifth and seventy-fifth percentiles; the solid line in the box is the median; the capped bars indicate the tenth and the ninetieth percentiles, and data points outside these limits are plotted as circles.

Slightly more *G. tachinoides*, *G. palpalis gambiensis* and *G. morsitans submorsitans* landed on biconical traps than on targets, but all differences were not significant (P>0.05; Figure 1 and Table 2). In contrast, landings were consistently lower on monoconical traps compared to targets for all three species, but only significantly lower for *G. tachinoides* (P<0.05; Figure 1 and Table 2). Sex ratios were similar on the different devices for the three species.

**Table 2:** Detransformed mean daily landings by *G. tachinoides*, *G. palpalis gambiensis* and *G. morsitans submorsitans* on biconical and monoconical traps and on targets.

Species	Trap type	Trap with adhesive film	Target with adhesive film	Catch index		P value
				trap	target	
*G. tachinoides*	bic	201.5	166.5	1.2		n/s
	mon	100.4	166.5		1.7	P<0.05
						
*G. p. gambiensis*	bic	30.0	24.5	1.2		n/s
	mon	15.3	24.5		1.6	n/s
						
*G. m. submorsitans*	bic	14.8	11.4	1.3		n/s
	mon	10.4	11.4		1.1	n/s

**bic** biconical trap, **mon** monoconical trap. The catch index is the proportion of the mean daily landings on the best device per row: n/s not significant (P>0.05) following ANOVA.

### Performance of POCA-baited trapping devices

The relative rankings of POCA-baited devices were very similar to those in the unbaited trials, with the target greatly outperforming the traps. Targets covered with adhesive film captured more than 10 times as many *G. tachinoides* as the biconical traps and about 50 times more than the monoconical traps (P≤0.001; Table 1), the traps not being covered by adhesive film. Baited targets also caught twice as many *G. palpalis gambiensis* as the biconical traps and 4–6 times more flies than the monoconical traps (P≤0.001; Table 1). For *G. morsitans submorsitans*, targets captured 8–9.5 times more flies than biconical traps and 20–22 times more than the monoconical traps (P≤0.001; Table 1).

The POCA bait did not affect the relative performance of the biconical over the monoconical trap for two species: the trapping rate of baited biconical traps was greater than monoconical traps made of the same material for *G. tachinoides* (P≤0.001) and *G. palpalis gambiensis* (P<0.05), except for the biconical in S250 blue for the latter species (Table 1). For *G. morsitans submorsitans*, POCA-baited biconical traps caught more than twice the number of flies as the monoconical traps (P≤0.01; Table 1). As in the unbaited trials, there was no difference between the performance of traps made from different blue cloths for any species (P>0.05) and sex ratios were similar on the different devices for the three species.

### Efficiency of biconical and monoconical traps

Trap efficiency, the proportion of flies landing which are then caught in the cage, has been estimated by dividing the mean daily catch of the unaltered biconical and monoconical traps by the mean daily catch of the matching traps with adhesive film on the cloth (flies caught on the adhesive film and in the cage; see Figure 1). The efficiency of the biconical trap varied between 11–22%, depending on the species. It was most efficient for *G. tachinoides* (22%) and *G. p. gambiensis* (21%) and only 11% efficient for *G. m. submorsitans* (Table 3). The efficiency of the monoconical trap was similar to the biconical trap, varying between 11–24%, most efficient for *G. p. gambiensis* (24%) and less efficient at trapping *G. m. submorstians* (14%) and *G. tachinoides* (11%; Table 3).

**Table 3:** Efficiency of biconical and monoconical traps for *G. tachinoides*, *G. palpalis gambiensis* and *G. morsitans submorsitans* calculated from detransformed mean daily catches.

Species	Trap type	Trap without adhesive film	Trap with adhesive film	Estimated trap efficiency %	% flies in cage of trap with film
*G. tachinoides*	bic	44.3	212.7	21%	7%
	mon	12.3	107.8	11%	8%
*G. p. gambiensis*	bic	7.0	31.9	22%	6%
	mon	4.6	19.5	24%	20%
*G. m. submorsitans*	bic	1.7	15.2	11%	2%
	mon	1.5	10.6	14%	2%

**bic** biconical trap, **mon** monoconical trap. The catch in the trap without adhesive film are the flies caught in cage of unaltered traps. The catch in the trap with adhesive film are the flies caught on the adhesive film and in the cage.

### Effects of adhesive film

Application of adhesive film to the target reduced the total number of *G. tachinoides* and *G. palpalis gambiensis* that apparently attempted to land on the target. The de-transformed catch indices for the two species compared to the unmodified target were 0.56 and 0.67, respectively (P≤0.01; Table 4), affecting both sexes equally. The effect of the adhesive film on fly behaviour nevertheless differed for the blue and black sections of the target, and between the two species, but not between the sexes. For *G. tachinoides*, the adhesive film had no effect on numbers landing on the blue section. In contrast, on the black section, addition of the adhesive film reduced catches of *G. tachinoides* by about two-thirds (P≤0.001; Table 5). For *G. palpalis gambiensis*, the adhesive film significantly increased the number of flies landing on the blue section (catch index 2.7). In contrast, on the black section, adhesive film reduced catches by about three-quarters (P≤0.001; Table 5). Field trials undertaken in Ethiopia on *G. tachinoides* using squares of transparent adhesive film alone as catching devices show that the film in itself when fixed vertically is not attractive (unpublished data).

**Table 4:** Detransformed mean daily catches of *G. tachinoides* and *G. palpalis gambiensis* on targets with and without adhesive film, expressed as a proportion of unmodified targets (catch index).

	Target	Target with adhesive film	Catch index
*G. tachinoides*	48.0	27.1	0.56***
*G. p. gambiensis*	19.5	13.0	0.67**

Catches on all targets were monitored with electric grids (see text).

Asterisks indicate that the indices are significantly different from unity:

**P≤0.01,

***P≤0.001 following Tukey post hoc test.

**Table 5:** Detransformed mean daily catches of *G. tachinoides* and *G. palpalis gambiensis* on the blue and black portions of targets with and without adhesive film, expressed as a proportion of unmodified targets (catch index).

	Blue			Black		
	Target	Target with adhesive film	Catch index	Target	Target with adhesive film	Catch index
*G. tachinoides*	15.0	15.4	1.0	32.4	11.0	0.3***
*G. p. gambiensis*	3.3	8.9	2.7**	16.9	4.4	0.26***

Catches on all targets were monitored with electric grids (see text).

Asterisks indicate that the indices are significantly different from unity:

**P≤0.01,

***P≤0.001 following Tukey post hoc test.

## Discussion

### Performance of targets versus traps

This study provides a cross-validation of the assumed efficiency of targets versus traps for the riverine tsetse species *G. tachinoides*, *G. palpalis gambiensis* and for the savannah species *G. morsitans submorsitans*. The number of flies landing on the outer surfaces of unbaited biconical or monoconical traps was not different to a standard 1×1 m target. This suggests that all of these blue-black objects provided adequate visual stimuli to attract tsetse, with differences in size, shape or contrast not critical to this key behaviour that underlies the efficacy of insecticide-impregnated control devices. Despite the fact that the greatest number of landings was recorded on the biconical trap for all three species, the differences compared to the two dimensional target are not significant. Only the blue material on the biconical trap was covered with adhesive film, and our results using electric-nets suggest that landing responses on the blue would have been unaffected or even slightly increased by the presence of the adhesive film. In contrast, results from the same trials show that the overall landing response on the target would have been reduced by 30–45% by the presence of the adhesive film, due to a reduced landing rate on the black material. This suggests that the landings recorded on the monoconical trap may also be slightly underestimated for the same reason, although the black cloth only accounts for a third of the potential landing area in cloth on this device. It therefore seems reasonable to assume that the landing response on biconical traps and targets without adhesive film would be more similar than the counts recorded here suggest, and if anything, probably even higher on the target.

The fabrication and maintenance of insecticide-impregnated cloth targets has obvious practical advantages over using traps, including being more economic. Laveissière et al. [17] in a study in Burkina Faso, showed that the relative efficacy of impregnated targets was initially similar to that of traps, but then fell with time. A similar result was obtained during early studies of targets in the Congo [18]. One possibility is that degradation of insecticide is lower in traps due to the shade provided by the trap cone or the trap body itself. After these initial comparisons, relevant tsetse control efforts for *G. tachinoides* and *G. palpalis gambiensis* using either impregnated traps [19], [20] or targets [21] have both achieved large reductions in tsetse population densities.

### Comparison of different trap and fabric types

Biconical traps consistently outperformed monoconical traps made of the same material for *G. tachinoides* and *G. palpalis gambiensis* in our trials. This is consistent with similar comparisons in Burkina Faso [22]. Laveissière and Grébaut [6] also found the same trends with *Glossina palpalis palpalis* in the Ivory Coast but recommended the monoconical trap because of its lower cost. All three blue materials tested (TDV phthalogen blue cotton, TDV phthalogen blue cotton/polyester, Sunflag Q10067 turquoise blue polyester/viscose) performed equally well in the same trapping device. These three blue fabrics would all be suitable for use in tsetse control devices provided that they are sufficiently colour-fast and have adequate insecticide retaining qualities. The similar performance of traps made from carefully matched fabrics suggests that effective targets can also be made from other modern phthalogen blue or turquoise blue fabrics that are dyed with variants of the pigment copper phthalocyanine [23]. This specific shade of blue was shown to be optimal for tsetse many years ago [24]. Similar care must be taken in selecting black fabrics that do not fade outdoors. Weathering properties and insecticide persistence need to be taken into account together when choosing fabrics that are optimal for long-term outdoor use as well as being attractive to tsetse [3]. The question also arises as to whether 100% cottons are as effective in retaining synthetic pyrethroids after field exposure as polyesters or blends. Assays with different fabrics and insects have not been consistent on this topic [25], [26]. Locally-made cotton targets have been used to suppress tsetse in Zimbabwe with excellent persistence of deltamethrin [27]. Hence, it is not clear why some tsetse control campaigns have chosen to use imported polyester targets instead of local cottons or blends [28], [29].

### Effect of POCA bait on trap and target performance

Importantly, trap entry/retention of flies did not appear to be improved by baiting traps with the POCA bait, i.e. baited targets also caught far more tsetse than baited traps. Baiting the devices with POCA did not affect their performance relative to one another, but appeared to increase the differences between them for *G. tachinoides.* The relative performance of traps for the three species remained the same, with the biconical trap always outperforming the monoconical trap. As the baited and unbaited trials were not simultaneous, they cannot be compared directly. However, similar experiments by Rayaisse et al. [30] on trap and target performance using electric nets showed that improvements in trap efficiency (i.e. greater than 2) can be achieved with this bait by up to a factor of 2 for *G. palpalis gambiensis* and up to a factor of 4 for *G. tachinoides*. Considering the efficacy of the targets, however, one should consider how much effort to invest in deploying and maintaining baits when it may be possible to adequately compensate for them by deploying more long-lasting inexpensive targets.

### Trap efficiency

As expected from many studies on savannah tsetse, both the biconical and monoconical traps were again found to be inherently inefficient as trapping devices for *G. tachinoides*, *G. palpalis gambiensis* and *G. morsitans submorsitans*, i.e. few flies that landed on attractive surfaces ended up being captured in the cage of the trap. Although the biconical trap attracts more flies than the monoconical trap and is the better landing device, the proportion of flies drawn to these trapping devices that get caught in the cage is similar for both traps types, varying between 11–24%. However, this efficiency of the two trap types is not always the same for an individual species, i.e. the trapping efficiency of the biconical and monoconical traps is similar for *G. p. gambiensis*, but the monoconical trap is only half as efficient as the biconical trap for *G. tachinoides*. Both types of trap are relatively inefficient at capturing *G. m. submorsitans* at the low fly densities recorded during these trials. An absolute interpretation of trapping efficiency is nevertheless difficult as there are many untested assumptions about fly behaviour and enumeration efficiency near traps that may affect results [31]. Bouyer et al. [32] have estimated that “long-range” biconical trap efficiency in Burkina Faso is only 1%; i.e. an unbaited trap catches only about 1 of 100 *G. palpalis gambiensis* present per km^2^ using mark-recapture techniques. In other words, within an area of 1 square km and over one day, only 1% of the flies are caught when using the biconical trap.

The trapping efficiencies of 11–22% for the biconical trap recorded here for *G. tachinoides* and *G. palpalis gambiensis* are similar in magnitude (8–33%) to those already measured in Burkina Faso for the same species in this trap type when it was assessed with a flanking electric net [30]. Although our experiments have shown that the use of adhesive film can reduce landing on the black elements of trapping devices, the consistency of the trapping efficiency estimates obtained for the biconical trap using either the adhesive film or electric nets validates the use of adhesive film as a measurement tool which would be particularly practical at the remote sites where some of these vectors are found.

### Concluding remarks

For the tsetse species studied here, the most efficient practical device for area-wide suppression would be a blue/black insecticide-impregnated target. For critical sampling, e.g. detecting residual pockets of tsetse in a complex landscape [33], the best device would clearly be a sticky target. Since this is not very practicable, biconical traps can be used but, in the light of tsetse fly densities encountered in this study, a correction factor dependent on population density (but of at least 5), needs to be applied to fly captures to compensate for the poor trapping efficiency of this device for the three species.
